# Diosmetin as an Effective Treatment for Cisplatin-Induced Neuropathic Pain in Mice which does not Cause Hepatic or Renal Biomarker Alterations

**DOI:** 10.1007/s12035-026-05785-0

**Published:** 2026-03-24

**Authors:** Patrick Tuzi Serafini, Rafaela Dias, Samuel Felipe Atuati, Sara Marchesan Oliveira

**Affiliations:** 1 Laboratory of Pain Neurobiology, Centre of Natural and Exact Sciences, Federal University of Santa Maria, Santa Maria, RS Brazil; 2 Graduate Program in Biological Sciences: Toxicological Biochemistry, Centre of Natural and Exact Sciences, Federal University of Santa Maria, Santa Maria, RS Brazil; 3 Department of Biochemistry and Molecular Biology, Centre of Natural and Exact Sciences, Federal University of Santa Maria, Camobi, Santa Maria, RS 97105-900 Brazil

**Keywords:** Flavonoids, Chemotherapy, Neuropathic pain, Nociceptive parameters, TRPV1 channels

## Abstract

Although cancer incidence is increasing globally, early detection and antineoplastic treatment ensure high patient survival rates. Among the chemotherapeutic agents widely used clinically, cisplatin is associated with the development of painful and non-painful peripheral neuropathy symptoms, compromising patient treatment continuity. Preclinical studies have indicated that the transient receptor potential vanilloid 1 (TRPV1) ion channels play a crucial role in chemotherapy-induced neuropathic pain. Currently, only duloxetine is recommended for treating chemotherapy-induced peripheral neuropathy, highlighting the urgent need for novel therapies. Natural products such as diosmetin have shown promising pharmacological potential. Here, we evaluated the effects of diosmetin in a mouse model of cisplatin-induced neuropathic pain (0.23 mg/kg, intraperitoneal). Nociceptive parameters included mechanical allodynia, affective-motivational behaviour, heat hyperalgesia, and muscle strength loss, along with hepatic [aspartate aminotransferase (AST), alanine aminotransferase (ALT)] and renal (urea and creatinine) biomarkers. Diosmetin (0.015, 0.15, and 1.5 mg/kg) administered orally by gavage (p.o.) resulted in antinociceptive effects, reduced mechanical allodynia, and decreased affective motivational behaviours in mice. Diosmetin (0.15 mg/kg) also reduced cisplatin-induced heat hyperalgesia and muscle strength loss, showing an efficacy comparable to that of duloxetine (30 mg/kg, p.o., positive control) and SB-366791 (1 mg/kg p.o., TRPV1 channel antagonist). Additionally, prior desensitisation of TRPV1-positive fibres with subcutaneous resiniferatoxin (RTX) prevents the development of mechanical allodynia and thermal hyperalgesia. Diosmetin (0.15 mg/kg) did not alter hepatic or renal biomarkers. Diosmetin has therapeutic potential for treating pain in patients with neuropathy, and the TRPV1 channel plays a crucial role in cisplatin-induced peripheral neuropathy.

## Introduction

The number of new cancer cases is expected to reach 35 million by 2050, representing a 77% increase compared to 2022 [1]. With the evolution of anticancer treatments and improved patient survival, greater attention is given to the potential adverse effects of these treatments, such as chemotherapy-induced peripheral neuropathy (CIPN), a common and serious neurological complication associated with the use of several antineoplastic agents [2–6]. CIPN affects up to 85% of patients undergoing treatment and is dependent on chemotherapy drugs and their respective doses [7–9]. The signs and symptoms of CIPN manifest as sensorimotor abnormalities, including burning pain, numbness, tingling, paraesthesia, distal weakness, spontaneous/ongoing pain, and hypersensitivity to mechanical and thermal stimuli [4, 5, 10].

Cisplatin, a platinum-based chemotherapeutic drug, is widely used in clinical anticancer therapy for metastatic solid tumours, including those of the lungs, bladder, ovaries, testes, neck, and head [9, 11]. Although cisplatin is an effective anticancer drug, its use is associated with neurotoxic effects on dorsal root ganglion (DRG) neurones, where it accumulates, causes DNA damage, and induces neuronal apoptosis [12–16]. Symptoms of cisplatin-induced peripheral neurotoxicity commonly manifest in both the upper and lower limbs during and after chemotherapy, severely affecting the daily activities of patients [11, 16]. Consequently, the neurotoxicity resulting from chemotherapy leads to dose reduction or treatment interruption in patients, negatively affecting their survival [2, 11, 17, 18].

Currently, there are no satisfactory pharmacological treatments for the symptomatic management of CIPN. Despite its limited efficacy, the antidepressant duloxetine has demonstrated clinical evidence of reducing both painful and non-painful symptoms of CIPN, making it the only pharmacological intervention recommended by the American Society of Clinical Oncology (ASCO) to treat CIPN [17, 19, 20]. Given the implications of chemotherapy in anticancer therapy and the scarcity of therapeutic options for managing CIPN symptoms, the development of novel therapeutic approaches remains a critical need. Thus, it is essential to develop innovative pharmacological strategies with favourable safety profiles that do not compromise the efficacy of antineoplastic agents.

To this end, it is essential to understand the underlying mechanisms by which many antineoplastic agents cause CIPN, which remain not fully understood [8, 12, 21, 22]. Chemotherapeutics can modulate the sensitivity and excitability of nociceptors in response to damage caused by G protein-coupled receptors and ion channels [22–27]. Moreover, preclinical studies have shown the crucial role of Transient Receptor Potential Vanilloid 1 (TRPV1) in pain symptoms induced by chemotherapeutic agents such as cisplatin and other antineoplastic drugs [28–36].

Diosmetin, a flavonoid of the flavone subclass found in a variety of plants and extracts of medicinal herbs [37], is a promising alternative for pain treatment, as it has shown antinociceptive activity in various pain models. Preclinical studies have demonstrated that diosmetin attenuates musculoskeletal and neuropathic pain induced by the antineoplastic agents anastrozole and paclitaxel, respectively, either individually or in combination. Diosmetin has also been shown to attenuate [38] neuropathic pain in a spinal nerve ligation model [39]. Additionally, diosmetin alleviated painful symptoms in reserpine-induced fibromyalgia and [40] ultraviolet B (UVB) radiation-induced sunburn models [41]. Although diosmetin is known to act as a TRPV1 antagonist, as it binds to TRPV1 in a specific binding assay, reduces capsaicin-induced calcium influx, and attenuates paw oedema and nociception caused by intraplantar capsaicin administration [42], the exact mechanism by which diosmetin alleviates painful symptoms in different pain models remains unclear. Therefore, since cisplatin modulates TRPV1 expression, and given the involvement of the TRPV1 channel in various neuropathic pain conditions, including those induced by chemotherapy, we tested the effect of diosmetin on cisplatin-induced painful symptoms [28, 30].

Here, we demonstrate that diosmetin exhibits antinociceptive effects in a mouse model of cisplatin-induced peripheral neuropathy. Our results, combined with previous findings, such as the absence of acute toxicity [42, 43] and the synergistic antitumour effect observed when diosmetin was associated with an antineoplastic drug [42–45], indicate that diosmetin is a promising candidate for the clinical treatment of peripheral neuropathic pain caused by chemotherapeutic drugs such as cisplatin.

### Drugs and Reagents

Cisplatin (cis-diaminedichloroplatinum II, C-Platin®; Blau, SP, Brazil) and mannitol (125 mg/kg) were diluted in 0.9% saline (0.9% NaCl). Diosmetin was purchased from Sigma-Aldrich Chemical Company (St. Louis, MO, USA) and dissolved in 1% dimethyl sulfoxide (1% DMSO) and 0.9% saline (99%). SB-366791 was purchased from Sigma-Aldrich Chemical Company (St. Louis, MO, USA) and was dissolved in a solution containing: (5%) tween® 80, (20%) polyethene glycol 400, and (75%) 0.9% saline. Duloxetine was purchased from Achè Laboratórios Farmacêuticos (Guarulhos, SP, Brazil) and dissolved in (10%) DMSO and (90%) 0.9% saline. Resiniferatoxin (RTX) was dissolved in (0.5%) ethanol, (0.5%) tween 80, (99%) and 0.9% saline. Ketamine and xylazine were purchased from Syntecvet (São Paulo, Brazil) and diluted in 0.9% saline. All other reagents, unless otherwise specified, were purchased from Sigma-Aldrich (St. Louis, MO, USA). The negative control group received vehicle in which the treatments were solubilised. Oral (by gavage, p.o.), subcutaneous (s.c.), and intraperitoneal (i.p.) treatments were administered to mice at a volume of 10 mL/kg.

### Animals

For the experiments, 126 male C57BL/6 mice (25–30 g; 8–10 weeks old) were provided by the Central Facility of the Federal University of Santa Maria (UFSM) and approved by the Institutional Animal Care and Use Committee (ethical process #7706310124) at the UFSM. Since other studies have shown no differences in the onset or severity of CIPN between male and female mice, we only evaluated the effects of cisplatin in male mice [46–49]. The animals were kept at a controlled temperature (22–23ºC), in a 12-h (h) light/dark cycle, with free access to water and food. The animals were handled in accordance with the standards of the National Council for the Control of Animal Experimentation (CONCEA), and the experiments were conducted in accordance with animal welfare standards and ethical considerations for the analysis of pain in conscious animals [50]. To minimise unnecessary pain and suffering, the number of animals and doses evaluated were kept to a minimum to demonstrate consistent effects of the procedures and treatments. The drug doses and experimental numbers used in this study were based on those used in previous studies [23, 26, 27, 51–53].

### Cisplatin-Induced Peripheral Neuropathy Model

**Fig. 1:**
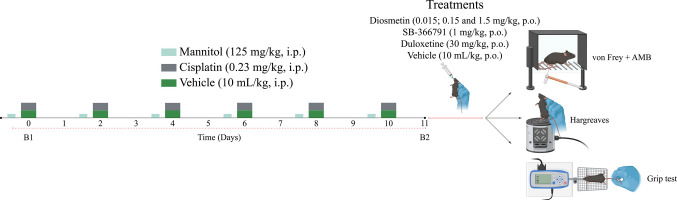
Experimental design: Peripheral neuropathy model induced by cisplatin, treatments, and behavioural tests. AMB: affective-motivational behaviour. Parts of Fig. 1 were elaborated from pictures provided by BioRender.com (https://www.biorender.com)

Cisplatin (0.23 mg/kg), or the vehicle (0.9% saline solution, 10 mL/kg) was administered intraperitoneally (i.p.) every 48 h for 10 days (days 0, 2, 4, 6, 8, and 10) [23, 26]. Mannitol (125 mg/kg, i.p.) was administered 1 h before cisplatin administration to prevent renal toxicity [23, 54]. The experimental design is illustrated in (Fig. 1).

### Study Design

#### Effect of Treatments on Nociceptive Parameters in Mice

**Fig. 2:**
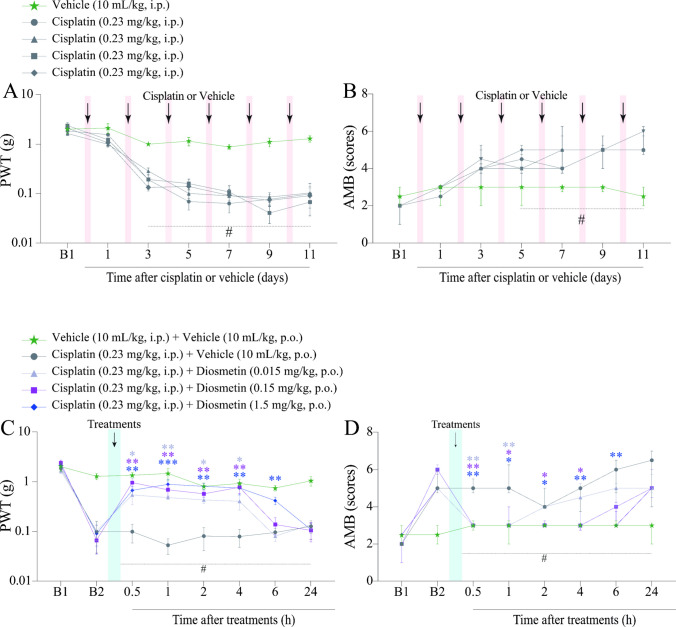
Cisplatin effect on the mechanical paw withdrawal threshold (PWT) and mechanical-induced affective-motivational behaviour (AMB) of mice. Male C57BL/6 mice were treated with intraperitoneal cisplatin or vehicle for a total of 6 doses over 10 days (**A** and **B**). Time- and dose–response curve of diosmetin on mechanical allodynia and mechanical-induced affective-motivational behaviour (AMB) caused by cisplatin (**C** and** D**). PWT and AMB were assessed on days 0 (Baseline 1; B1), 1, 3, 5, 7, 9, and 11 (Baseline 2; B2 in A and B), respectively. PWT and AMB were evaluated on day 11 (B2), and from 0.5 to 24 h after diosmetin or its vehicle treatments (**C** and **D**). B1 values ​​were measured before cisplatin or vehicle administration. B2 values ​​were measured on day 11 after the first cisplatin dose and before oral treatments. The values of B1 in A and B are the same as the B1 represented in C and D, respectively. The measurements on day 11 in A and B are equivalent to the measurements of B2 in C and D, respectively. PWT and AMB were evaluated in the same animals. Parametric data (mechanical allodynia) are expressed as mean ± SEM (*n* = 6); two-way ANOVA followed by Bonferroni *post hoc test*. Nonparametric data (AMB) are expressed as medians with interquartile ranges and analysed by the Kruskal–Wallis test followed by Dunn's* post hoc test* (*n* = 6). ^#^*P* < 0.05 when compared with vehicle group; ^*^P < 0.05, ^**^P < 0.01 and ^***^P < 0.001 when compared with cisplatin + vehicle group. SEM: standard error of the mean

**Fig. 3:**
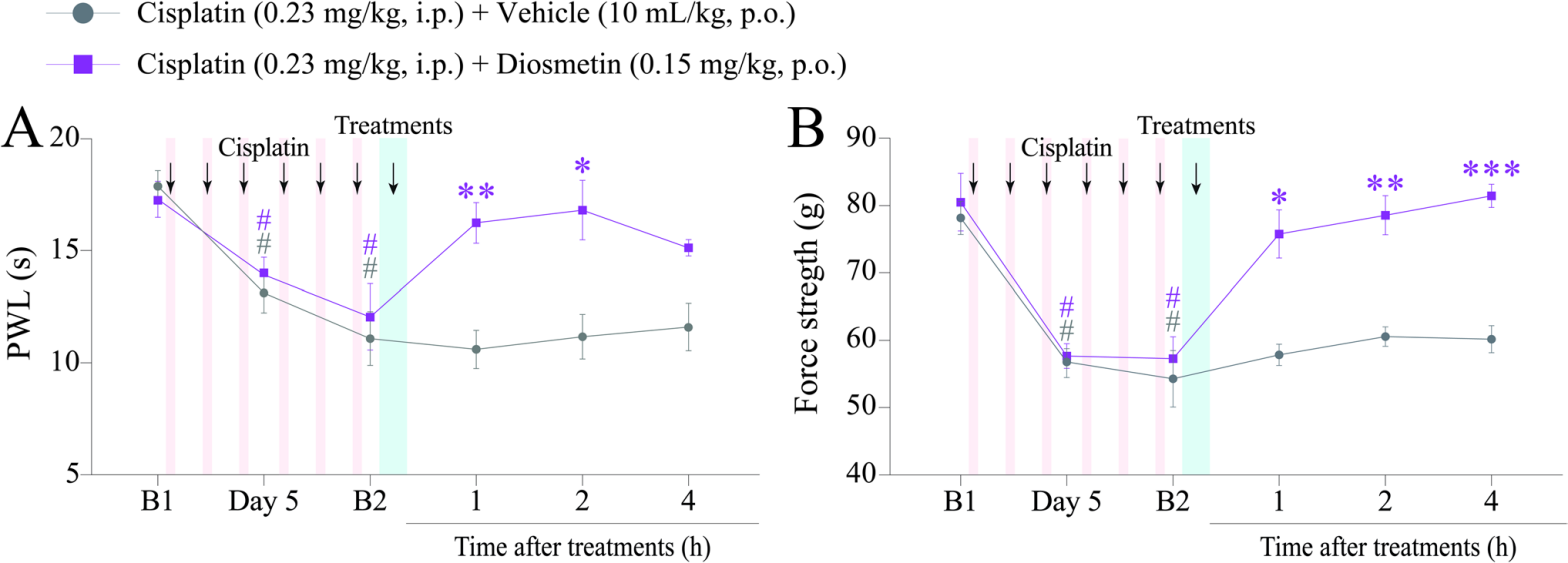
Time-response curve of the diosmetin on heat hyperalgesia and muscle strength loss induced by cisplatin. (**A** and **B**) Male C57BL/6 mice were treated with intraperitoneal cisplatin for a total of 6 doses over 10 days. (**A** and **B**) Thermal paw withdrawal latency (PWL) and muscle strength were assessed on days 0 (Baseline 1; B1), 5, and 11 (Baseline 2; B2), respectively. On day 11 (B2), the same parameters were assessed again from 1 to 4 h after treatment. B1 values ​​were measured before cisplatin administration. B2 values ​​were measured on day 11 after the first cisplatin dose and before oral treatments. Parametric data are expressed as mean ± SEM (*n* = 6); two-way ANOVA followed by Bonferroni *post hoc test*. PWT and AMB were evaluated in the same animals. ^#^*P* < 0.05 when compared with baseline values ​​(B1); ^*^P < 0.05, ^**^P < 0.01 and ^***^P < 0.001 when compared with cisplatin + vehicle group. SEM: standard error of the mean

The mechanical paw withdrawal threshold (PWT) and mechanically induced affective-motivational behaviour (AMB) were measured in mice on day 0 (baseline 1; B1) and on days 1, 3, 5, 7, 9, and 11 (baseline 2; B2) after the first cisplatin administration (Fig. 1; Fig. 2A and B). On the 11th day (B2) after the first cisplatin administration, the animals received diosmetin (0.015, 0.15 and 1.5 mg/kg, p.o.) or its vehicle [(99%) saline solution 0.9% + (1%) DMSO; 10 mL/kg, p.o.], and the mechanical PWT and AMB were again evaluated at 0.5, 1, 2, 4, 6, and 24 h after these treatments (Fig. 1; Fig. 2C and D). As described in the Results section, since diosmetin at doses of 0.15 and 1.5 mg/kg reduced similarly nociceptive parameters assessed, we selected the diosmetin intermediate dose (0.15 mg/kg) to adopting the 3Rs principle and minimise the total number of animals used in the study. Time intervals from 1 to 4 h were employed to perform the muscle strength tests (Grip test) and thermal hypersensitivity to heat test (Hargreaves test) (Fig. 1; Fig. 3A and B).

**Fig. 4:**
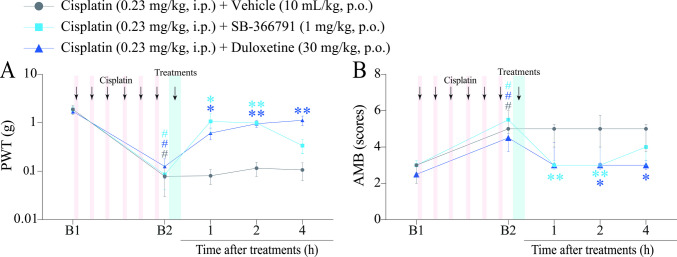
Time-response curve of SB-366791 and duloxetine on mechanical allodynia and mechanical-induced affective-motivational behaviour (AMB) caused by cisplatin. (**A** and **B**) Male C57BL/6 mice were treated with intraperitoneal cisplatin for a total of 6 doses over 10 days. (**A** and **B**) Mechanical paw withdrawal threshold (PWT) and AMB were assessed on days 0 (Baseline 1; B1) and 11 (Baseline 2; B2), respectively. On day 11, the same parameters were evaluated from 1 to 4 h after treatments. B1 values ​​were measured before cisplatin administration. B2 values ​​were measured on day 11 after the first cisplatin dose and before oral treatments. Parametric data (mechanical allodynia) are expressed as mean ± SEM (*n* = 6); two-way ANOVA followed by Bonferroni *post hoc test*. Nonparametric data (AMB) are expressed as medians with interquartile ranges and analysed by the Kruskal–Wallis test followed by Dunn's *post hoc test* (*n* = 6). PWT and AMB were evaluated in the same animals. ^#^*P* < 0.05 when compared with B1 values; ^*^P < 0.05 and ^**^P < 0.01 when compared with cisplatin + vehicle group. SEM: standard error of the mean

**Fig. 5:**
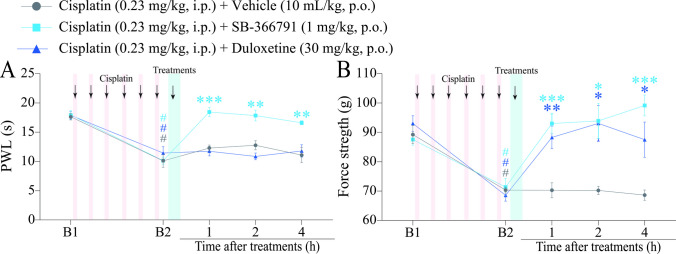
Time-response curve of SB-366791 and duloxetine on heat hyperalgesia and muscle strength loss induced by cisplatin. (**A** and **B**) Male C57BL/6 mice were treated with intraperitoneal cisplatin for a total of 6 doses over 10 days. (**A** and **B**) Paw withdrawal latency (PWL) and muscle strength were assessed on days 0 (Baseline 1; B1) and 11 (Baseline 2; B2), respectively. On day 11, the same parameters were evaluated from 1 to 4 h after treatments. B1 values ​​were measured before cisplatin administration. B2 values ​​were measured on day 11 after the first cisplatin dose and before oral treatments. PWL and muscle strength were evaluated in the same animals. Parametric data are expressed as mean ± SEM (*n* = 6); two-way ANOVA followed by Bonferroni *post hoc test*. ^#^*P* < 0.05 when compared with B1 values;^*^P < 0.05, ^**^P < 0.01 and ^***^P < 0.001 when compared with cisplatin + vehicle group. SEM: standard error of the mean

The effect of the reference drug duloxetine (30 mg/kg, p.o.), the TRPV1 antagonist, SB-366791 (1 mg/kg, p.o.) or its vehicle [(5%) tween® 80 + (20%) polyethylene glycol 400 + (75%) saline solution 0.9%, v.o.] was also evaluated on the 11th day (B2) after the first cisplatin dose (Fig. 1). In this protocol, we assessed the same nociceptive parameters as previously mentioned, using the same time intervals from 1 to 4 h, to evaluate the antinociceptive effects of the reference drug duloxetine and the TRPV1 channel antagonist SB-366791 (Fig. 1; Fig. 4A and B; Fig. 5A and B).

#### Effect of TRPV1-Positive Fibres Desensitisation Induced By Resiniferatoxin

We also evaluated whether cisplatin could induce neuropathic pain symptoms in animals previously treated with RTX, which promotes the desensitisation of TRPV1-positive nerve fibres [55–57].

**Fig. 6:**
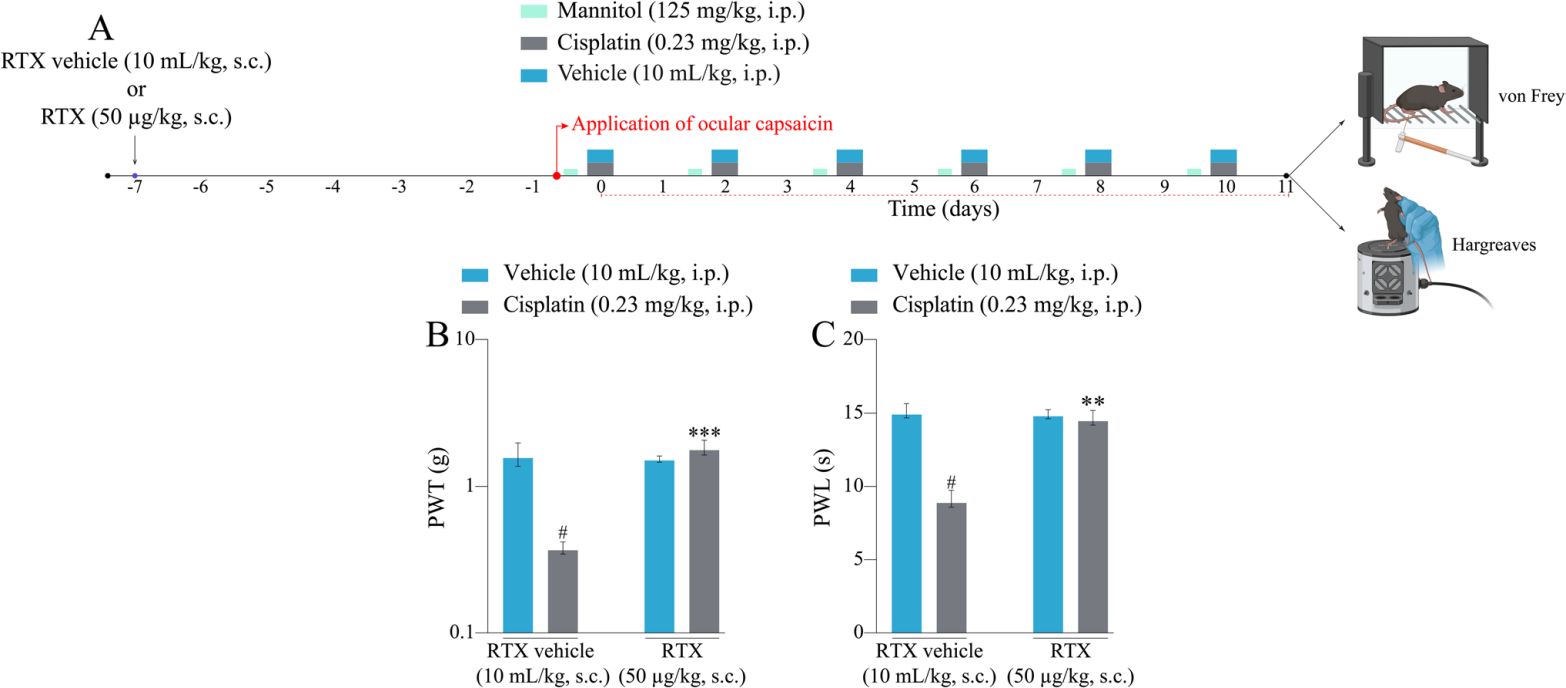
Effect of systemic resiniferatoxin (RTX; 50 µg/kg, s.c.) administration on cisplatin-induced mechanical allodynia and heat hyperalgesia in mice. (**A**) Male C57BL/6 mice were treated with intraperitoneal cisplatin or vehicle for a total of 6 doses over 10 days. (**B** and **C**) Mechanical paw withdrawal threshold (PWT) and thermal paw withdrawal latency (PWL) were assessed in a single measurement on the 11th day after the first cisplatin dose, respectively. PWT and PWL were evaluated in different animal groups. Parametric data are expressed as mean ± SEM (*n* = 6); one-way ANOVA followed by Dunnett’s T3 multiple-comparisons test. ^#^P < 0.05 compared with vehicle + RTX vehicle group; ^**^P < 0.01 and ^***^P < 0.001 compared with cisplatin + RTX vehicle group. SEM: standard error of the mean

First, the animals were anaesthetised intraperitoneally with ketamine (90 mg/kg) and xylazine (3 mg/kg) to minimise pain and discomfort during RTX (50 µg/kg, s.c.) administration [55–57]. Subsequently, RTX (50 µg/kg, s.c.) or its vehicle (0.9% saline, 10 mL/kg, s.c.) was administered in a single dose (Fig. 6A). On the 7th day after RTX administration, we performed a topical ocular application test using 5 µL of capsaicin (1 nmol/5 µL = 0.2 nmol/µL), verifying the cleaning attacks for 1 min after ocular application of capsaicin, to confirm the desensitisation of the TRPV1 channel. The absence of cleaning attacks after the application of capsaicin eye drops in animals treated with RTX was considered a positive result of TRPV1 channel desensitisation.

Topical ocular application was performed only in animals subjected to the desensitisation protocol but not in the vehicle. After confirming TRPV1 channel desensitisation, the animals received cisplatin protocol (0.23 mg/kg, intraperitoneally) every 48 h for 10 days. Mechanical allodynia and thermal hypersensitivity were measured on the 11th day after the first cisplatin administration (Fig. 6A).

### Nociceptive Parameters

#### Mechanical Paw Withdrawal Threshold and Affective-Motivational Behaviour

The mechanical PWT of mice was assessed using flexible nylon (von Frey) filaments of increasing stiffness (0.02–10 g) according to the "up-and-down" method [58] (Fig. 1). Animals were placed on an elevated platform with a metal mesh bottom that allowed full access to the plantar surface of the paws. Filaments were applied to the right hind paw of the mice with sufficient force to deform them into a "C" shape, for a total of six applications. The filament with an intermediate force (0.4 g) was applied first. In the event of a negative response to the application of the filament, we used the subsequent filament with the greatest force in grams (g). Conversely, in the case of a positive response, we used the subsequent filament with the lowest force (g). If four consecutive (positive or negative) responses occurred, we cease applying the filaments.

The mechanical PWT 50%, expressed in grams, was calculated from thresholds obtained with Frey filaments, as described in previous studies, [59, 60] using the appropriate software [58, 61]. Mechanical allodynia was defined as a decrease in mechanical PWT relative to baseline (B1) values ​​before or after cisplatin administration or relative to the vehicle group.

Mechanically induced AMB was assessed according to previous studies [41], with some modifications (Fig. 1). Affective-motivational responses were assessed alongside the mechanical PWT (to the same extent). To approximate the defensive responses to perceptual experiences of pain, the AMB test was used to evaluate each animal's individual perception of the stimulus [62]. After the application of each von Frey filament (which can vary from 0.02 to 10 g), the animals' behavioural responses were observed, and a score was assigned based on their behaviours: 0 = no response; 1 = rapid paw shaking; 2 = paw licking; 3 = paw withdrawal; 4 = escape jump or hyperlocomotion. The results are expressed as the sum of the scores (from six applications) for each animal.

#### Paw Withdrawal Latency to Heat

The paw withdrawal latency (PWL) of the mice to the heat stimulus was assessed using a radiant thermal stimulus (60 W radiant light in the Hargreaves apparatus) applied to the right hind paw (Fig. 1) [63]. To avoid damage to the paw tissue, the maximum exposure time to the radiant heat source was 20 s [60]. The PWL was assessed from 1 to 4 h to avoid sensitisation of the plantar tissue. Hyperalgesia to heat was defined as a reduction in PWL in seconds (s) relative to baseline values ​​(B1) during or after cisplatin administration or in the vehicle group.

#### Muscle Strength Assessment

Muscle strength was assessed using grip testing (grip test device) (Fig. 1) [52]. The animals were placed on a metal grid connected to a grip strength meter and gently pulled from their tails until they were released. The maximum force exerted by the animal’s paws was recorded in grams using the device. The measurements were analysed in triplicate with a 1-min interval between each test [24]. A reduction in muscle strength was considered when the applied grip force (in grams) decreased relative to the baseline values ​​(B1) during or after cisplatin administration or in the vehicle group.

### Biochemical Markers

#### Measurement of AST and ALT Enzyme Activity and Serum Urea and Creatinine Levels

At the end of the experiments, on the 11th day after the first dose of cisplatin, the animals were deeply anaesthetised with sodium thiopental (150 mg/kg, i.p.), and blood was collected by cardiac puncture. The samples were then centrifuged at 3000 rpm for 10 min at room temperature, and the serum obtained was used to determine the levels of aspartate aminotransferase (AST) and alanine aminotransferase (ALT) using the Labtest colourimetric kit. AST and ALT levels were measured at 340 nm, and the results are expressed as U/L.

Serum samples were also used to measure urea and creatinine levels using the Labtest kit. The results are expressed as milligrams per millilitre (mg/mL) for urea and milligrams per decilitre (mg/dL) for creatinine.

### Statistical Analysis

Results are expressed as mean ± standard error of the mean (SEM). All statistical analyses were performed using GraphPad Prism version 9.5.0 (GraphPad Software, USA). Differences between groups were analysed using two-way analysis of variance (ANOVA), with F values ​​indicating the interaction between time and treatment (column) factors, or one-way ANOVA with Brown-Forsythe correction, as appropriate. Bonferroni and Dunnett’s T3 post hoc multiple comparison tests were applied following one- or two-way ANOVA when indicated. The Geisser-Greenhouse correction was used to adjust the degrees of freedom for the F-tests when the sphericity assumption was violated. PWT data were logarithmically transformed to meet parametric assumptions. Nonparametric data (AMB scores) were analysed using the Kruskal–Wallis test, followed by Dunn's post hoc test. Inhibition percentages were calculated by comparing the maximal responses with those of the corresponding control groups. Differences were considered statistically significant at *P* < 0.05.

## Results

### Diosmetin Attenuates Cisplatin-Induced Mechanical Allodynia and Affective-Motivational Behaviour

We evaluated the antinociceptive effect of diosmetin (0.015, 0.15 and 1.5 mg/kg, p.o.) using time- and dose–response curves in mice subjected to cisplatin-induced painful peripheral neuropathy (Fig. 1A). First, we demonstrated that cisplatin caused mechanical allodynia in mice from the 3rd to 11th day (B2) after the first cisplatin dose compared to the vehicle group [F (24, 150) = 4.637,* P* < 0.05; (Fig. 2A)]. All tested doses of diosmetin alleviated mechanical allodynia in mice compared with the cisplatin + vehicle group [F (28, 175) = 6.955, *P* < 0.05; Fig. 2C]. Diosmetin at doses of 0.015 and 0.15 mg/kg reduced mechanical allodynia from 0.5 to 4 h after treatment, when compared to the cisplatin + vehicle group, with inhibitions of 76.71 ± 6.15% (at 2 h) and 93.17 ± 8.29% (at 4 h), respectively. The 1.5 mg/kg dose reduced mechanical allodynia from 0.5 to 6 h, reaching 100% inhibition at 2 h.

We also evaluated the effect of diosmetin on mechanically induced affective motivational behaviour (AMB) in mice subjected to the same protocol (Fig. 1). Cisplatin increased the AMB of mice from the 5th to 11th day (B2) after the first cisplatin dose, compared to the vehicle group (Fig. 2B). Diosmetin at doses of 0.015 and 0.15 mg/kg decreased AMB from 0.5 to 1 h and from 0.5 to 4 h after treatment, respectively, compared with cisplatin + vehicle group, with 92.30 ± 7.69% inhibition at 0.5 h (Fig. 2D). Diosmetin at 1.5 mg/kg decreased AMB from 0.5 to 6 h, reaching 100% inhibition at 6 h) after treatment compared with the cisplatin + vehicle group (Fig. 2D). Because the antinociceptive effects of diosmetin 0.15 and 1.5 mg/kg reduced nociceptive parameters similarly, we selected the intermediate diosmetin dose of 0.15 mg/kg, for subsequent testing.

### Diosmetin Attenuates Cisplatin-Induced Heat Hyperalgesia and Muscle Strength Loss

We also evaluated the effect of diosmetin (0.15 mg/kg, p.o.) on the painful parameters of heat hyperalgesia and muscle strength loss in mice subjected to cisplatin-induced painful peripheral neuropathy (Fig. 1). Cisplatin caused heat hyperalgesia and muscle strength loss on days 5th and 11th (B2) after the first dose compared with the baseline values ​​(B1). Diosmetin (0.15 mg/kg) reduced the cisplatin-induced heat hyperalgesia at 1 and 2 h after treatment, reaching 100% inhibition at 1 h [F (5, 50) = 5.252, *P* < 0.05] (Fig. 3A). The same dose also attenuated muscle strength loss from 1 to 4 h, with complete inhibition at 2 and 4 h [F (5, 60) = 6.304,* P *< 0.05] (Fig. 3B), compared with the cisplatin + vehicle group.

The experimental results did not provide data to identify the molecular pathways that mediate the antinociceptive effects of diosmetin. However, they demonstrated a potential antinociceptive effect of diosmetin on cisplatin-induced peripheral neuropathy (Fig. 2C and D; Fig. 3A and B).

### SB-366791 and Duloxetine Alleviate Cisplatin-Induced Mechanical Allodynia and Affective-motivational Behaviour

We also evaluated the antinociceptive action of the TRPV1 channel antagonist SB-366791 (1 mg/kg, p.o.) and duloxetine (30 mg/kg, p.o.), the reference drug, in cisplatin-induced painful peripheral neuropathy (Fig. 1). Cisplatin induced mechanical allodynia on day 11 (B2) after the first cisplatin dose compared with baseline values ​​(B1). SB-366791 and duloxetine alleviated the mechanical allodynia of mice compared to the cisplatin + vehicle group [F (8, 60) = 4.224, *P* < 0.05; Fig. 4A]. SB-366791 (1 mg/kg) reduced the mechanical allodynia at 1 and 2 h (with inhibition of 81.90 ± 4.04% at 2 h) after treatment when compared to the cisplatin + vehicle group. Similarly, duloxetine (30 mg/kg) reduced mechanical allodynia from 1 to 4 h after treatment (with an inhibition of 81.53 ± 10.90% at 4 h) compared with the cisplatin + vehicle group.

We also evaluated the development of mechanically induced AMB in mice with cisplatin-induced painful peripheral neuropathy (Fig. 1). Cisplatin increased the AMB of mice on day 11 (B2) after the first cisplatin dose compared to baseline values (B1). SB-366791 (1 mg/kg) decreased AMB at 1 and 2 h (with inhibition of 84.61 ± 11.91% at 1 h) after treatment when compared to the cisplatin + vehicle group (Fig. 4B). Similarly, duloxetine (30 mg/kg) attenuated AMB at 2 and 4 h (with inhibition of 93.75 ± 6.24% at 2 h) after treatment when compared to cisplatin + vehicle (Fig. 4B).

### SB-366791 Alleviates Heat Hyperalgesia and Muscle Strength Loss, While Duloxetine Attenuates Only cisplatin-induced Muscle Strength Loss

We also evaluated the effect of the TRPV1 channel antagonist SB-366791 (1 mg/kg, p.o.) and duloxetine (30 mg/kg, p.o.), a reference drug, on the pain parameters of heat hyperalgesia and muscle strength loss in cisplatin-induced painful peripheral neuropathy mice (Fig. 1). Cisplatin induced heat hyperalgesia and muscle strength loss on day 11 (B2) after the first cisplatin dose when compared to baseline values ​​(B1). SB-366791 (1 mg/kg), but not duloxetine (30 mg/kg), reduced the cisplatin-induced heat hyperalgesia (from 1 to 4 h with inhibition of 100% at 1 and 2 h) after treatment when compared to cisplatin + vehicle [F (8, 60) = 6.533,* P* < 0.05; Fig. 5A]. Both SB-366791 (1 mg/kg) and duloxetine (30 mg/kg) reduced muscle strength loss from 1 to 4 h (with 100% inhibition from 1 to 4 h for SB-366791 and at 2 h for duloxetine) after treatment when compared to the cisplatin + vehicle group [F (8, 60) = 6.838, *P* < 0.05; Fig. 5B].

### TRPV1 Desensitisation By Resiniferatoxin Prevents Cisplatin-induced Mechanical Allodynia and Heat Hyperalgesia

We also evaluated whether cisplatin is capable of inducing mechanical allodynia and heat hyperalgesia in mice previously treated with RTX (50 µg/kg, s.c.) (Fig. 6A). Cisplatin induced mechanical allodynia [F (3, 10.5) = 13.9,* P* < 0.05; Fig. 6B] and heat hyperalgesia [F (3, 17.5) = 16.8; *P* < 0.05; Fig. 6C] in the RTX vehicle group compared to the vehicle + RTX vehicle group. RTX administration did not alter the mechanical or thermal thresholds compared to those in the vehicle + RTX vehicle group. In contrast, RTX administration prevented the development of mechanical allodynia and heat hyperalgesia induced by cisplatin compared with the cisplatin + RTX vehicle group. Desensitisation of TRPV1-positive fibres was confirmed by topical ocular application of 5 µL capsaicin (1 nmol/5 µL), as these mice did not exhibit cleaning attacks during the observed period.

### Measurement of Biochemical Markers in Serum Samples

We assessed whether diosmetin (0.15 mg/kg, administered orally) affected the biochemical markers AST, ALT, urea, and creatinine in serum samples obtained via cardiac puncture. The findings indicated that neither cisplatin nor diosmetin (acute dose) altered the serum levels of AST, ALT, urea, or creatinine compared to the control group (vehicle + vehicle) (Table 1). These results support the safety profile of diosmetin (0.15 mg/kg) when given acutely, showing no evidence of renal or hepatic toxicity and confirming an acceptable safety profile.

**Table 1:** Effect of acute diosmetin treatment on serum AST, ALT, urea, and creatinine levels

Groups	AST (U/L)	ALT (U/L)	Urea (mg/mL)	Creatinine (mg/dL)
Vehicle (10 mL/kg, i.p.) + Vehicle (10 mL/kg, p.o.)	42.16 ± 10.48	14.02 ± 2.88	30.87 ± 7.55	0.90 ± 0.10
Cisplatin (0.23 m/kg, i.p.) + Vehicle (10 mL/kg, p.o.)	64.50 ± 7.34	8.74 ± 1.26	50.73 ± 12.67	0.97 ± 0.05
Cisplatin (0.23 m/kg, i.p.) + Diosmetin (0.15 m/kg, p.o.)	45.83 ± 9.06	11.72 ± 1.52	37.03 ± 7.36	0.83 ± 0.08

Male C57BL/6 mice were treated with 6 doses of cisplatin or vehicle intraperitoneally over 10 days. On the 11th day after the first cisplatin administration or its vehicle, the animals were treated with diosmetin or its vehicle. At the end of the experiments, blood was collected by cardiac puncture to obtain serum. Data are expressed as mean ± SEM (*n* = 6), one-way ANOVA followed by Bonferroni *post-hoc* test. *SEM* Standard error of the mean, *i.p.* intraperitoneal, *p.o.* oral route

## Discussion

Cancer is one of the leading causes of death before the age of 70 years in 112 countries [64], and chemotherapeutic drugs remain the primary treatment tool for a wide range of cancers [22]. However, its use is associated with chemotherapy-induced peripheral neuropathy (CIPN), necessitating new, safe, and effective treatments [4, 5, 22]. Here, we show that diosmetin attenuates cisplatin-induced pain parameters, including mechanical allodynia, mechanically induced affective-motivational behaviour, heat hyperalgesia, and muscle strength loss. Although the mechanism by which diosmetin attenuates these nociceptive parameters remains to be elucidated, the antinociceptive effect of diosmetin was comparable to that of the reference drug, duloxetine, and the TRPV1 channel antagonist, SB-366791. Furthermore, we showed that the desensitisation of TRPV1-positive nerve fibres with RTX prevented the development of mechanical allodynia and heat hyperalgesia in cisplatin-treated mice, confirming the involvement of TRPV1 channels, a possible target of diosmetin, in this cisplatin-induced peripheral neuropathy model. Acute diosmetin administration did not cause renal or hepatic alterations in the animal serum samples.

Cisplatin-induced peripheral toxicity is both time- and dose-dependent, and painful peripheral neuropathy is a dose-limiting and frequent adverse effect of platinum compounds in clinical use [11]. In our preclinical mouse model of cisplatin-induced painful peripheral neuropathy, we reproduced the pain symptoms frequently reported in patients treated with cisplatin [11, 17]. Cisplatin causes mechanical allodynia, heat hyperalgesia, reduced muscle strength, and increased mechanically induced AMB in mice. The latter represents a negative defensive reflexive behaviour that initiates the animal's motivation to cease aversive sensations, involving limbic circuits associated with the basolateral amygdala. The act of licking or withdrawing the affected limb from the applied stimulus aims to reduce the aversive qualities involving the individual affective-motivational components of each animal processed in the limbic and cortical circuits of the brain [62, 65]. To the best of our knowledge, this is the first study to demonstrate that cisplatin increases AMB behaviour in mice in the CIPN model. In addition, cisplatin induced other painful symptoms (mechanical allodynia, heat hyperalgesia, and muscle weakness), similar to a wide range of previous preclinical studies, in which cisplatin induced peripheral neuropathy, [23, 26, 28, 30, 66–76] and in an experimental model of muscle atrophy in which cisplatin caused a loss in grip strength in mice [77–79]. The cisplatin dose used in our study did not alter renal and hepatic biomarkers. Our data agree with those of Becker et al. [23], in which cisplatin at a tenfold higher dose (2.3 mg/kg) than that used in our study did not cause renal or hepatic damage in mice.

Diosmetin reduces cisplatin-induced mechanical hypersensitivity, AMB, heat hyperalgesia, and loss of muscle strength in mice. However, the molecular mechanisms by which diosmetin relieves cisplatin-induced pain were not investigated in our study. To date, previous studies suggest that some mechanistic pathways are involved in the diosmetin's antinociceptive effects, which include regulation of the Keap1/Nrf2/NF-κB signalling pathway, and modulation of the p38 mitogen-activated protein kinase (MAPK)-dependent pathway and pro-inflammatory cytokine levels [39, 80–84], as well possible interaction with the TRPV1 channel [42]. TRPV1 channels detect a wide range of stimuli and play a crucial role in pre- and postsynaptic neurones; therefore, TRPV1 has been implicated in the pathogenesis of CIPN [10, 85–88]. In this context, preclinical trials have demonstrated the TRPV1 involvement in mechanical allodynia and heat hyperalgesia, as well as in muscle strength loss in peripheral neuropathy models induced by cisplatin or other classes of chemotherapy drugs [28–30, 73, 78, 89–91]. Therefore, TRPV1 is a key contributor in the perception and modulation of neuropathic pain [88, 92]. Furthermore, in a previous study, diosmetin demonstrated antinociceptive effects, regardless of sex, in neuropathic pain induced by spinal cord injury, through regulation of the Keap1/Nrf2/NF-κB signalling pathway [39], which could be another pharmacological target of diosmetin to alleviate the cisplatin-induced nociceptive parameters.

Another pharmacological target of diosmetin for alleviating these symptoms may be the mitogen-activated protein kinase (MAPKs) pathway, which is an important mediator of intracellular signalling in the transduction of pain stimuli [93]. p38 MAPK, a protein kinase belonging to the MAPK family, has been implicated in neuronal apoptosis induced by cisplatin [30, 94], which is consistent with the ability of cisplatin to induce p38 phosphorylation [30, 67, 94]. Phosphorylated p38 activates secondary messengers that sensitise protein kinases and phosphorylate ion channels [30]. Diosmetin modulates intracellular p38 MAPK-dependent pathways, alleviates acute lung injury induced by the H1N1 influenza virus, induces the maturation and differentiation of human osteoblastic cells [83, 95], and exerts anti-inflammatory and antioxidant effects against endotoxin-induced acute liver failure [82].

In the context of neuropathic pain, such as that caused by chemotherapy, and owing to the role of neuroinflammation in neuropathy, flavonoids have shown promise as alternatives owing to their anti-inflammatory properties [96, 97]. Diosmetin can decrease pro-inflammatory cytokine levels (IL-1β, IL-6, and TNF-α) [39, 80, 81, 84], which are key mediators of cisplatin-induced neuropathic pain [28, 30, 98]. Although all the mechanisms described above may contribute to the antinociceptive effect of diosmetin, further studies are needed to elucidate how diosmetin reduces cisplatin-induced pain. Considering that CIPN involves multiple mechanisms, the effects of diosmetin may stem from both the previously described and unknown mechanisms. A limitation of our study is the lack of investigation into alternative mechanisms that could expand the pathways through which diosmetin exerts its antinociceptive effects.

In addition to neuroinflammatory mechanisms, alterations in ion channels, such as TRPV1, have been implicated in the symptoms associated with painful peripheral neuropathy [10, 88]. Thus, it is important to note that TRPV1 plays a critical role in thermal pain detection [86], as well as in the detection of pain from mechanical stimuli in CIPN [88], which explains the attenuation of heat hyperalgesia, mechanical allodynia, and mechanically induced AMB, by SB-366791. Additionally, TRPV1 antagonism by SB-366791 could explain the recovery of mouse muscle strength, as TRPV1 exerts pro-nociceptive effects through sensory fibres that innervate both the skin and muscle tissues [99]. According to this evidence, subcutaneous administration of TRPV1 agonists, such as capsaicin and RTX, caused musculoskeletal hyperalgesia and consequent loss of muscle strength in mice, as assessed by the grip force assay [100]. This effect was prevented by SB-366791, supporting the involvement of TRPV1 channels in muscle pain modulation [100]. Our results are consistent with those of previous studies showing the antinociceptive action of SB-366791 [51, 89, 101] in clinically relevant pain models, including neuropathic and [38, 42, 101] reserpine-induced nociplastic pain models [40, 51].

To reinforce the role of TRPV1 channels in cisplatin-induced peripheral neuropathy, we performed functional desensitisation using a single dose of the ultrapotent TRPV1 agonist, RTX, which suppressed the excitability of peripheral neurons and their central projections [102]. Here, desensitisation of TRPV1-expressing fibres prevented the development of cisplatin-induced mechanical allodynia and heat hyperalgesia. These data corroborate studies in which desensitisation of TRPV1 with RTX prevented reserpine-induced mechanical allodynia and heat hyperalgesia in fibromyalgia [51] and spinal nerve ligation-induced neuropathic pain models [102]. Furthermore, silencing TRPV1 also prevented cisplatin-induced mechanical allodynia and heat hyperalgesia [73]. These results confirmed that TRPV1 is involved in the nociceptive symptoms caused by cisplatin in the CIPN experimental model and corroborated the critical TRPV1 role in neuropathic pain.

The mechanisms of chronic pain are complex, involving not only peripheral and central sensitisation but also alterations in descending pain-modulation pathways [103]. In our study, duloxetine, a dual serotonin and norepinephrine reuptake inhibitor, reduced mechanical allodynia, mechanically induced AMB, and muscle weakness. Our results are consistent with those of a wide range of studies reporting that duloxetine reduces these parameters [38, 104–110], possibly by increasing the efficacy of the descending inhibitory pain pathway [109]. In contrast, duloxetine did not reduce cisplatin-induced thermal hyperalgesia, similar to the results of another study on peripheral neuropathy caused by repeated paclitaxel administration [38]. By contrast, Wang et al. [109] reported that duloxetine relieved paclitaxel-induced thermal hyperalgesia. The discrepancy between our results and those reported by Wang et al. [109] may have resulted from the inhibition of TRPV1 upregulation by repeated duloxetine treatment, whereas we administered a single treatment. Clinically, duloxetine is the only treatment recommended by the ASCO for the relief of symptoms associated with chemotherapy [17]. However, duloxetine demonstrated moderate efficacy, with 59% of patients reporting pain reduction and 41% reporting an improvement in numbness and tingling symptoms in the feet [19]. In clinical practice, duloxetine causes adverse effects such as dry mouth, insomnia, dizziness, and fatigue, resulting in a high treatment discontinuation rate of 54.8% in cases of prolonged use [19]. Therefore, there is an urgent need for novel, safe, and effective therapies for chemotherapy-associated neuropathic pain.

Preclinical data suggest a favourable safety profile for diosmetin in the treatment of painful pathological conditions. Previous studies have demonstrated that diosmetin 0.15 mg/kg neither alters body temperature, weight, and locomotor activity (spontaneous and forced), nor alters markers of liver or kidney injury, and does not induce ulcerogenic activity or modify gastrointestinal transit in experimental animals [40, 42]. Moreover, an acute toxicity study with diosmetin at 300 and 2000 mg/kg did not reveal any histopathological changes in the kidneys or liver, nor did it alter the behaviour or phenotype of the animals treated with diosmetin during the experiments [43]. We also showed that diosmetin did not cause hepatotoxicity or nephrotoxicity under the experimental conditions evaluated. However, preclinical studies are required to analyse the safety profile of long-term administration. In this context, the antinociceptive effect of diosmetin following long-term administration should assess the impact of repeated diosmetin doses and exclude the possible development of analgesic tolerance. Importantly, for patients undergoing chemotherapy, where the predominant adverse effect is the development of painful peripheral neuropathy, preventing the development of painful symptoms is essential. Therefore, future studies should evaluate the effects of diosmetin treatment to prevent or attenuate the development of chemotherapy-induced pain symptoms.

A limitation of our study was the lack of data supporting the antinociceptive effect of diosmetin in female mice with chemotherapy-induced neuropathy. Future studies should evaluate whether sex interferes with the antinociceptive effects of diosmetin in chemotherapeutic drug-induced neuropathic pain models. Furthermore, because cold sensitivity is a characteristic sensory manifestation of cisplatin-induced peripheral neuropathy [2, 9], future investigations should evaluate the effects of diosmetin on this sensory abnormality.

## Conclusion

We demonstrated that diosmetin exhibited antinociceptive effects in cisplatin-induced painful neuropathy without signs of acute toxicity, and that its efficacy was similar to that of SB-366791, a selective TRPV1 antagonist, and duloxetine, a reference drug, reinforcing its clinical relevance in CIPN. The crucial role of TRPV1 in cisplatin-induced pain symptoms was confirmed by channel desensitisation with RTX. Despite the relevance of TRPV1 in the pathophysiology of neuropathic pain, our results are insufficient to determine a direct role of TRPV1 as a pharmacological target of diosmetin. In this context, the antinociceptive effects of diosmetin are likely mediated by multifactorial mechanisms. The lack of information on how diosmetin binds to or functionally regulates the TRPV1 channel is a limitation of our study and requires further investigation. Taken together, our results indicate that diosmetin is a promising alternative for treating painful CIPN. Further studies are needed to elucidate the molecular mechanisms of diosmetin, as well as its detailed safety and bioavailability profile, including its effects after long-term administration.

## Data Availability

All data generated in this study are available upon reasonable request to the corresponding author.

## Abbreviations

AMB: Affective-motivational behaviour
ANOVA: Analysis of variance
ASCO: American Society of Clinical Oncology
CIPN: Chemotherapy-induced peripheral neuropathy
DMSO: Dimethylsulfoxide
DRG: Dorsal Root Ganglion
I.P.: Intraperitoneal
MAPKs: Mitogen-activated protein kinases
P.O.: Oral route
PWL: Paw withdrawal latency
PWT: Paw withdrawal threshold
RTX: Resiniferatoxin
SEM: Standard error of the mean
S.C.: Subcutaneous
TRPV1: Transient Receptor Potential Vanilloid 1
UVB: Ultraviolet B

## Declarations

### Credit Images

Figure 1 was elaborated from pictures provided by BioRender.com (https://www.biorender.com).
